# Combined intensive management of fertilization, tillage, and organic material mulching regulate soil bacterial communities and functional capacities by altering soil potassium and pH in a Moso bamboo forest

**DOI:** 10.3389/fmicb.2022.944874

**Published:** 2022-08-25

**Authors:** Ying Zheng, Xinzhu Liu, Yanjiang Cai, Qingsong Shao, Wei Zhu, Xinchun Lin

**Affiliations:** ^1^State Key Laboratory of Subtropical Silviculture, Zhejiang A&F University, Hangzhou, China; ^2^Zhejiang Provincial Key Laboratory of Resources Protection and Innovation of Traditional Chinese Medicine, Zhejiang A&F University, Hangzhou, China; ^3^Protection of Ecological Forestry Research Center in Huzhou, Huzhou, China

**Keywords:** intensive management, *Nitrospirae*, denitrification, soil chemical property, Moso bamboo, microbiota

## Abstract

Intensive management is a common practice in agricultural and forestry ecosystems to improve soil quality and crop yield by influencing nutrient supply and soil microbiota; however, the linkage between soil nutrients and bacterial community and functional capacities in intensively managed economic forests has not been well studied. In this study, we investigated the soil properties such as available potassium (AK), available nitrogen (AN), available phosphorus (AP), ammonium (NH${}_{4}^{+}$), nitrate (NO${}_{3}^{-}$), organic matter (OM), total nitrogen (TN), total phosphorus (TP), bacterial diversity and community composition, potential functions of rhizome roots, and soil microbiota across a chronosequence of intensively managed Moso bamboo (*Phyllostachys edulis*) forests. Our results demonstrated that the combined intensive management (deep tillage, fertilization, and organic material mulching) in this study caused a significant increase in the concentrations of AK, AN, AP, NH${}_{4}^{+}$, NO${}_{3}^{-}$, OM, TN, and TP (*P* < 0.05). However, they led to a remarkable decrease in pH (*P* < 0.05). Such changes lowered the Shannon diversity of the soil and rhizome root microbiota but did not significantly affect the community composition and functional capacity. Soil bacterial community variation was predominantly mediated by soil total potassium (TK) (15.02%), followed by pH (11.29%) and AK (11.13%). We further observed that *Nitrospirae* accounted for approximately 50% of the variation in soil pH, NO${}_{3}^{-}$, NH${}_{4}^{+}$, and AK, indicating its importance in soil nutrient cycling, especially nitrogen cycling. Accordingly, we propose that the management-induced changes in soil parameters reshaped the bacterial community structure and keystone bacterial assemblage, leading to the differentiation of microbial functions.

## Introduction

To enhance the sustainable development of forest resources, intensive management, a widely used practice in agricultural ecosystems, is now frequently adopted in forest ecosystems with targets to improve soil productivity and forest products. It can alter soil biological characteristics, prevent soil nutrient loss, control crop pests and diseases, and affect soil quality and sustainability (Geisseler and Scow, 2014; Lin et al., 2018; Siedt et al., 2021). The improvement of plant yield under intensive management is closely related to the abundance of bacterial communities in the soil (Wang et al., 2020a). As the core drivers of soil nutrient cycling, soil bacterial microorganisms contribute substantially to the stability of soil properties (Zhang et al., 2020), but are prone to management-induced disturbances. A better understanding of soil properties and bacterial communities is fundamental for optimizing management practices and improving soil quality for sustainable forest ecosystem production (Chen et al., 2019; Li et al., 2020a; Liu et al., 2021). However, the effects of intensive forest management on soil bacterial communities and their associated functional capacity are poorly understood (Colombo et al., 2016).

Soil bacterial diversity and community composition are sensitive to intensive management and respond differently to various types of management practices. Fertilization, mulching, and tillage are the three most common management practices, among which fertilization is the most frequently and widely applied (Li et al., 2020b; Man et al., 2021; Yang et al., 2021). Applying an appropriate amount of fertilizers can improve plant growth and productivity by providing nutrients for plants and soil microorganisms; however, excessive fertilization leads to a nutrient surplus, producing adverse effects on plant growth (Huang et al., 2021). Kong et al. (2019) reported that long-term fertilization altered soil nitrification efficiency by affecting active autotrophic ammonia and nitrite oxidizers. Continuous nitrogen (N) fertilization in rice paddies increased soil nitrification rates and nitrate availability, which could eventually increase denitrification (Pandey et al., 2018, 2019). Mulching practices can alter bacterial diversity and community composition by improving the physical environment of the soil, such as soil temperature and moisture (Wang et al., 2020c; Zhang et al., 2020; Gabriel et al., 2021). Therefore, changes in soil properties during mulching may induce the reconstruction of bacterial assemblages, affecting bacterial community structure and function (Dong et al., 2017; Qian et al., 2018). Tillage, a moderate form of disturbance, temporarily enhances soil aeration, releases organic matter, and creates new ecological niches for soil microorganisms (Degrune et al., 2017). Conversing to conservation tillage enriched nitrogen-cycling bacterial communities in sandy soils under a long-term maize monoculture. Subsoiling tillage with straw incorporation improved subsoil and topsoil microbial community characteristics (Liu et al., 2022) and metabolic activity (Denier et al., 2022).

Unlike agricultural and other forestry plants, bamboos are mainly clonal and characterized by extremely long intervals between flowering periods (7–120 years) and a highly complicated rhizome root network (Clark et al., 2015; Wysocki et al., 2016). Moso bamboo (*Phyllostachys edulis*) is the most widely distributed bamboo species in subtropical China, accounting for 72.96% of the total bamboo forest area (Li and Feng, 2019). This bamboo species provides numerous economic benefits through timber and shoots' production and plays a major role in atmospheric carbon sequestration and soil erosion control in degraded areas owing to rapid biomass accumulation and well-developed rhizome systems (Zhou et al., 2011). To promote the early emergence of edible bamboo shoots, combined intensive management, namely, inorganic fertilizer application, deep tillage, and organic material (straw + bamboo leaves + rice chaff) mulching, has been frequently practiced in recent years, particularly in the Zhejiang province, China (Chen et al., 2019; Cao et al., 2020). Combined intensive management practices yield much earlier bamboo shoots and a higher market price (20–40 RMB kg^−1^ vs. 0.6–1 RMB kg^−1^) than regular management with only fertilization and tillage applied (Jiang et al., 2006). Therefore, combined intensive management largely increases the income of farmers, reduces economic stress, and promotes local economic development (Cao et al., 2014). However, long-term intensive management has resulted in adverse effects, such as edaphic quality decline, bamboo forest recession, and environmental degradation (Jiang et al., 2000; Gui et al., 2013; Xu et al., 2017). Fallow has been suggested for application in Moso bamboo forests to alleviate the bamboo forest recession induced by intensive management. Nonetheless, the effects of combined intensive management and fallow on the bacterial community and functional capacities in Moso bamboo forests remain unclear.

A better understanding of the diversity, composition, and biological functions of soil bacteria under combined intensive management would be conducive to taking adequate measures to promote the sustainable development of forestry ecosystems. In the present study, a high-throughput paired-end Illumina sequencing method was applied to study the bacterial diversity, community composition, and functional capacities of Moso bamboo rhizome roots and soil microbiota at three soil depths (0–10 cm; 10–20 cm; 20–30 cm) under combined intensive management. We sought to explore the nature and alterations in the bacterial diversity, community composition, and population dynamics under combined intensive management. We further explored the mechanisms by which combined intensive management affects the functional capacities of soil bacterial microbiota.

## Materials and methods

### Site description and experimental design

**Figure 1:**
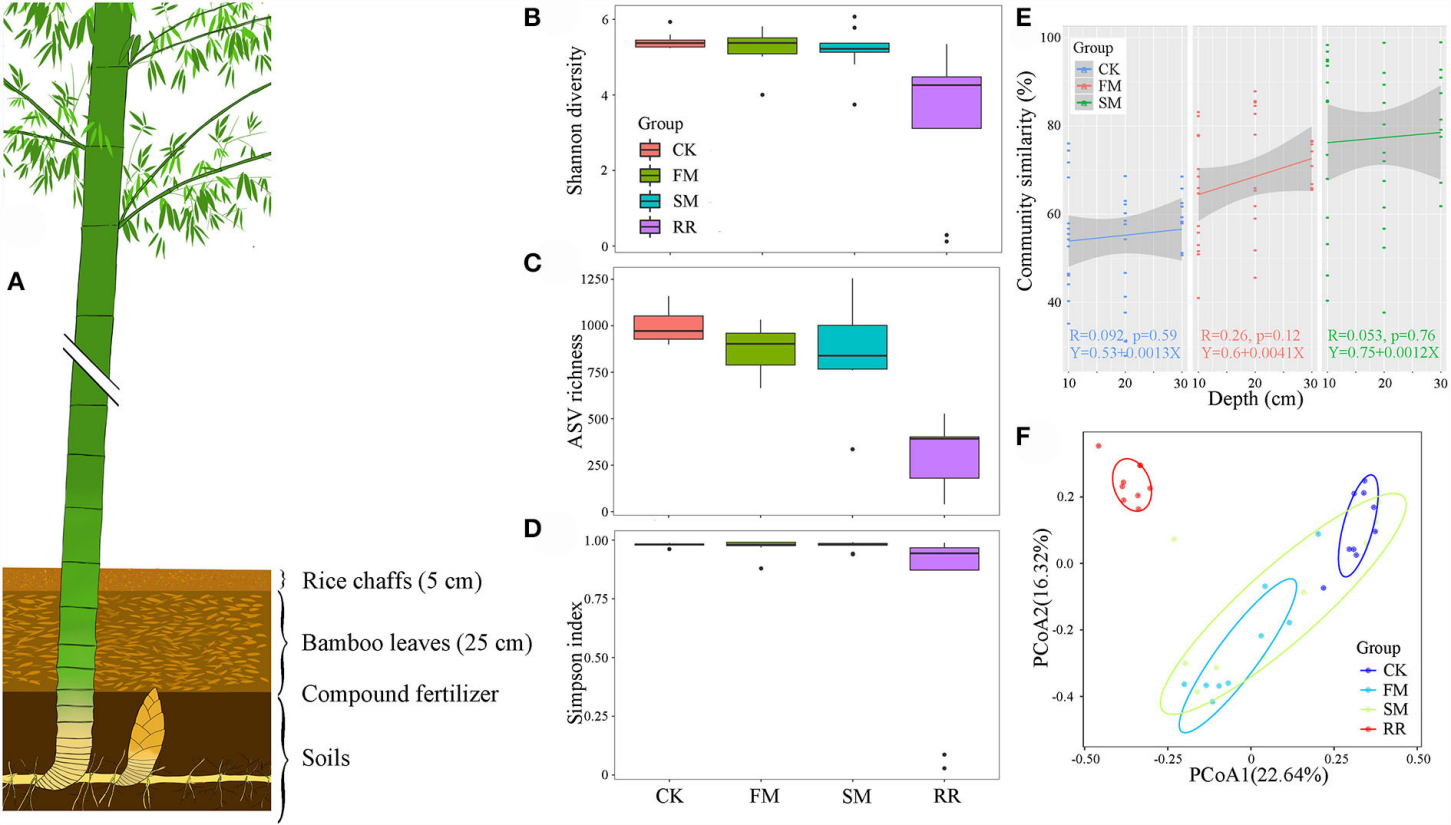
A diagram of combined intensive management of Moso bamboo forests **(A)** and its effect on the diversity and composition of Moso bamboo rhizome roots and soil bacteria. **(B)** Shannon diversity, **(C)** ASV richness, and **(D)** Simpson index were calculated with 999 permutations in R *v*. 3.6.1 (R Core Team, 2019). Box plots showed the range of estimated values between 25% and 75%, the median, the minimum, and the maximum observed values within each dataset. **(E)** The similarity of soil bacterial communities under different management periods across soil depth. The lines denote the least-squares linear regressions across soil depth, with 95% confidence intervals (gray-shaded areas). **(F)** Principal coordinates analysis (PCoA) of bacterial community composition among different groups based on the Bray–Curtis dissimilarity matrix performed by the online tool iSanger (https://cloud.majorbio.com). Ellipses cover the data for each group.

The experiments were conducted in a Moso bamboo forest in Huzhou, Zhejiang province, China (30°46'39”N, 120°00'34”E). This region is characterized by a subtropical monsoon climate with an annual average temperature of 15.6°C, annual precipitation of 1,309 mm, and annual sunshine duration of 1,810.3 h. The soil is siltstone with a thick soil layer and little gravel. Three treatments were assigned to each plot in a randomized block design with three replicates. The block size of each plot was 3 × 3 m. Intensive management was conducted in November with an average temperature of approximately 10°C. The detailed processes are listed as follows: (1) before practice, the soil was well watered and then turned over to a depth of 30–40 cm. (2) Compound fertilizer (N:P:K = 1:1:1) was applied as base fertilizer at a rate of 0.15 kg m^−2^ and then covered by 25 cm Moso bamboo leaves, and 5 cm rice chaffs were subsequently added on the top. (3) The chaffs were removed the following year, from March to early April, when few bamboo shoots emerged. (4) Additional 0.15 kg m^−2^ compound fertilizers were applied and turned over with the remaining bamboo leaves to the soils (Figure 1A). The first treatment was conducted continually with the abovementioned practices for 2 years, named “FM.” The second treatment was intensively managed for 3 years, followed by fallow for the following 3 years, and finally highly practiced for another 2 years (SM). The third group was a control without combined intensive management (CK).

### Sample collection and processing

Bamboo shoots were randomly selected from each block, and their rhizome roots (RR) were collected on 21 January 2019. Bulk soils surrounding the bamboo shoots were collected at depths of 0–10, 10–20, and 20–30 cm. In total, 27 (3 plots × 3 blocks × 3 depths) soil samples and nine rhizome roots (3 plots × 3 blocks) were obtained. Visible plant roots, stones, litter, and debris were removed from soil samples and separated into two subsamples. One subsample was stored at −80°C for DNA isolation and the other was air-dried for soil chemical property analysis. Rhizome roots were kept separately in sterile tubes, transferred to the laboratory as soon as possible, and stored at −80 °C for DNA isolation. Soil and plant relicts adhering to the surface of the rhizome roots were manually removed under running water. The samples were kept in running water for 1–2 h and surface-sterilized for 2 min in 75% ethanol. Subsequently, the samples were washed three times with distilled water and sterilized again with 2.5% sodium hypochlorite for 10 min using a vacuum filter pump, followed by another five washes with distilled water. Sterility was assessed by placing 100 μL of the last washing water on Luria–Bertani (LB) agar plates for 3–7 days cultivated in a 28°C incubator. Samples that did not exhibit bacterial growth on LB plates were used for DNA extraction.

### DNA extraction and sequencing

The total genomic DNA of the rhizome roots, namely, the host DNA and its endophytes, was extracted using a modified cetyltrimethylammonium bromide (CTAB) method. Soil DNA was extracted from 0.5 g soil samples using the MoBio PowerSoil^TM^ DNA isolation kit (MoBio Laboratories, CA, USA) according to the manufacturer's instructions. Primer sets 799F and 1193R were used to amplify the V5–V7 region of the bacterial 16S rRNA gene (Bulgarelli et al., 2012). The PCR amplification was performed in a 20-μL reaction mixture containing 10 ng of template DNA, 0.4 μL of TransStart FastPfu DNA Polymerase, 4 μL of 5 × FastPfu Buffer, 2 μL of dNTP (2.5 mM), and 0.8 μL of each primer (5 μM). After an initial denaturation step at 95°C for 3 min, the targeted region was amplified by 27 cycles of 95°C for 30 s, 55°C for 30 s, and 72°C for 45 s, followed by a final elongation step of 10 min at 72°C using the 799F and 1392R primers. The second-step primers were 799F-1193R, and identical conditions to the first step of the PCR were applied for 15 cycles. PCR products were analyzed using 2% agarose gel electrophoresis. Amplicon libraries and DNA sequencing were conducted using an Illumina MiSeq PE300 platform by Shanghai Majorbio Bio-Pharm Technology Co., Ltd., following the manufacturer's protocols. The DNA sequences were deposited in the National Center for Biotechnology Information BioProject database under BioProject accession ID: PRJNA692804 (https://submit.ncbi.nlm.nih.gov/subs/sra/SUB8903000/overview).

### Soil chemical property analyses

Soil pH was determined in a 1:2.5 w v^−1^ soil-to-water suspension using a pH electrode (Mettler Toledo, Switzerland). Soil organic matter (OM) content was determined by potassium dichromate oxidation (K_2_Cr_2_O_7_), and total nitrogen (TN) was estimated using the semi-micro-Kjeldahl method. Alkalytic N (AN) was analyzed using the diffusion method. Available phosphorus (AP) was determined using the Bray procedure (Bray and Kurtz, 1945), total phosphorus (TP) (Wakelin et al., 2008) was determined using the microwave digestion method, total potassium (TK) was measured using a flame photometer, and available potassium (AK) was measured using the flame photometric method. Ammonium nitrogen (NH${}_{4}^{+}$) and nitrate nitrogen (NO${}_{3}^{-}$) were extracted from the soils with a 1 M KCl solution (1:10 w v^−1^) for 1 h, and their concentrations were determined using a continuous flow autoanalyzer (Skalar, Breda, the Netherlands). All the methods mentioned earlier were described by Lu (1999).

### Bioinformatics analysis of 16S rRNA gene profiling

Bioinformatics processing of 16S rRNA sequences was based on previous studies (Zheng et al., 2018b; Zhang et al., 2019; Zheng and Gong, 2019; Zheng and Lin, 2020) using QIIME (Caporaso et al., 2010), USEARCH (Edgar, 2010), and in-house scripts. Briefly, after generating high-quality reads, unique reads were assigned to amplicon sequence variants (ASVs). The taxonomy of the representative ASVs was classified using the RDP classifier (Wang et al., 2007) based on the SILVA 132 database (Quast et al., 2012). The R package “EasyAovWlxPlot” was applied to calculate the significant differences in soil prosperities among treatments (R Core Team, 2019). Aggregated boosted tree (ABT) modeling was used to estimate the relative influence of soil prosperities on the abundance of soil microbiota after mulching (De'ath, 2007).

### Statistical analysis

Statistical analyses were performed using R and STAMP software. The normal distribution of the data was checked with the Shapiro–Wilk test, and the homogeneity of variances was analyzed using Levene's test in R with the “car” package (Fox and Weisberg, 2019). The analysis of variance (ANOVA) was performed to verify whether significant differences in microbial diversity existed among different management practices (Rinke et al., 2013). Spearman's correlation coefficients were calculated to identify the relationships between the microbial parameters and physicochemical properties of the soil. Furthermore, multiple pairwise comparisons between the mean of groups at a 95% family-wise confidence level were conducted based on Tukey's honestly significant difference test (Tukey's HSD). Principal coordinates analysis (PCoA) of the microbial community structure was performed based on the Bray–Curtis dissimilarity matrix using the online tool iSanger (https://cloud.majorbio.com) with the “Pearson” distance measure and “ward” clustering algorithm. Permanova and pairwise comparisons were conducted using the “adonis” and “pairwise.adonis” functions with the “bray” method and 10,000 iterations by the “vegan” package in R (Oksanen et al., 2019). Additionally, Venn diagrams displaying the overlap of ASVs revealed by different data analysis methods were plotted using BIOINFOGP (Oliveros, 2007). Differential ASV abundance and taxa were analyzed using the Wilcoxon rank sum test at both the ASV and family levels. The corresponding *P*-values were corrected for multiple tests using a false discovery rate (FDR) set at 0.05. Functional prediction and metabolic characteristics of representative ASVs were carried out using PICRUSt (Langille et al., 2013) and FAPROTAX (Louca et al., 2016) with significantly different catalogs between soil and root microbiomes estimated in STAMP with the Kruskal–Wallis H test and Welch's *t*-test corrected by Bonferroni (Parks et al., 2014).

## Results

### Soil chemical properties

Soil NH${}_{4}^{+}$, AK, NO${}_{3}^{-}$, AN, OM, TP, TN, and AP were significantly higher in FM and SM than in CK (Table 1). The contents of soil TP, TN, AK, NO${}_{3}^{-}$, AN, and AP increased, and the contents of OM and NH${}_{4}^{+}$ decreased in SM compared to that in the FM. Notably, performing FM significantly decreased the soil pH; however, after fallow for 3 years with SM, no significant difference in soil pH was observed between SM and CK.

**Table 1:** Soil chemical properties in the Moso bamboo forests under combined intensive management.

**Group**	**AK (mg kg^−1^)**	**AN (mg kg^−1^)**	**AP (mg kg^−1^)**	**NH${}_{4}^{+}$ (mg kg^−1^)**	**NO${}_{3}^{-}$(mg kg^−1^)**	**OM (g kg^−1^)**	**TK (g kg^−1^)**	**TN (g kg^−1^)**	**TP (g kg^−1^)**	**pH**
CK	109.58 ± 15.60a	0.06 ± 0.01a	14.10 ± 25.10a	16.86 ± 2.54a	1.64 ± 0.64a	13.90 ± 4.01a	28.30 ± 2.23a	6.22 ± 1.14a	0.31 ± 0.10a	4.88 ± 0.17a
FM	415.91 ± 62.71b	0.13 ± 0.05b	52.57 ± 42.00b	46.84 ± 27.75b	47.71 ± 31.06b	30.02 ± 13.94b	29.15 ± 1.85a	12.50 ± 7.05b	0.55 ± 0.18b	4.23 ± 0.17b
SM	713.17 ± 269.52b	0.15 ± 0.04b	206.88 ± 122.63c	35.58 ± 12.53b	69.79 ± 85.18b	28.41 ± 10.05b	20.15 ± 1.38b	12.91 ± 3.51b	1.03 ± 0.39c	4.94 ± 0.42a

Data are presented as mean ± standard deviation (n = 3). Different lowercase letters within a column indicate statistically significant differences between treatments at P = 0.05 level. AK, available potassium; AN, available nitrogen; AP, available phosphorus; NH${}_{4}^{+}$, ammonium; NO${}_{3}^{-}$, nitrate; OM, organic matter; TK, total potassium; TN, total nitrogen; TP, total phosphorus.

### Bacterial diversity and composition of soil and rhizome root microbiota

#### Bacterial diversity of soil and rhizome root microbiota

A total of 851,701 high-quality 16S rDNA sequences (548,049 from soil samples and 303,652 from rhizome root samples) were retained after quality trimming and chimera checking, with an average read length of 375 bp. After removing the potential chloroplast, mitochondrial, and eukaryotic sequences, 2,677 amplicon sequence variants (ASVs: 2,409 from soil samples and 1,437 from rhizome root samples) were obtained and assigned to different taxonomic levels using the SILVA classifier. All rarefaction curves tended to reach a plateau, indicating that the sequencing sample size was sufficient and that the sequencing data were suitable for downstream analysis.

A significant difference in alpha diversity indices (namely, Shannon diversity, ASV richness, and Simpson index) was observed between the soil and root bacterial microbiota, but not among the intensive practices (Figures 1B–D). Specifically, the Shannon diversity and richness were remarkably higher in the soil microbiota than in the root microbiota (ANOVA, *P* < 0.05). All alpha diversity indices of soil bacteria decreased in response to combined intensive management. With the SM practice, the Shannon diversity of soil bacteria decreased compared to that of FM (Figure 1B), whereas the ASV richness increased (Figure 1C). The Simpson index of soil bacteria remained relatively stable during combined intensive management (Figure 1D).

The ADONIS analysis revealed that sample types (soils vs. rhizome roots) and combined intensive management affected bacterial communities, but not soil depths. We further compared the vertical spatial variation in each bacterial community group down through the soil depth profiles among different management times to assess the ADONIS results. The vertical spatial decay relationship (VDR) slopes of all bacterial groups were steepest in FM (R = 0.26). The bacterial VDR slope of the SM was the smallest (R = 0.053). But the difference in community similarity among different practices was not significant (*P* > 0.05, Figure 1E). Therefore, we mainly focused on the bacterial comparison at the sample type and combined intensive management levels.

For PCoA, the first and second principal coordination axes accounted for 22.64 and 16.32% of the total variation among different samples, respectively (Figure 1F). Rhizome roots exhibited a significant difference in bacterial composition compared to that in the soil samples. The bacterial composition of the soil microbiota did not vary significantly after different intensive practices.

#### Composition of soil and root bacterial community

**Figure 2:**
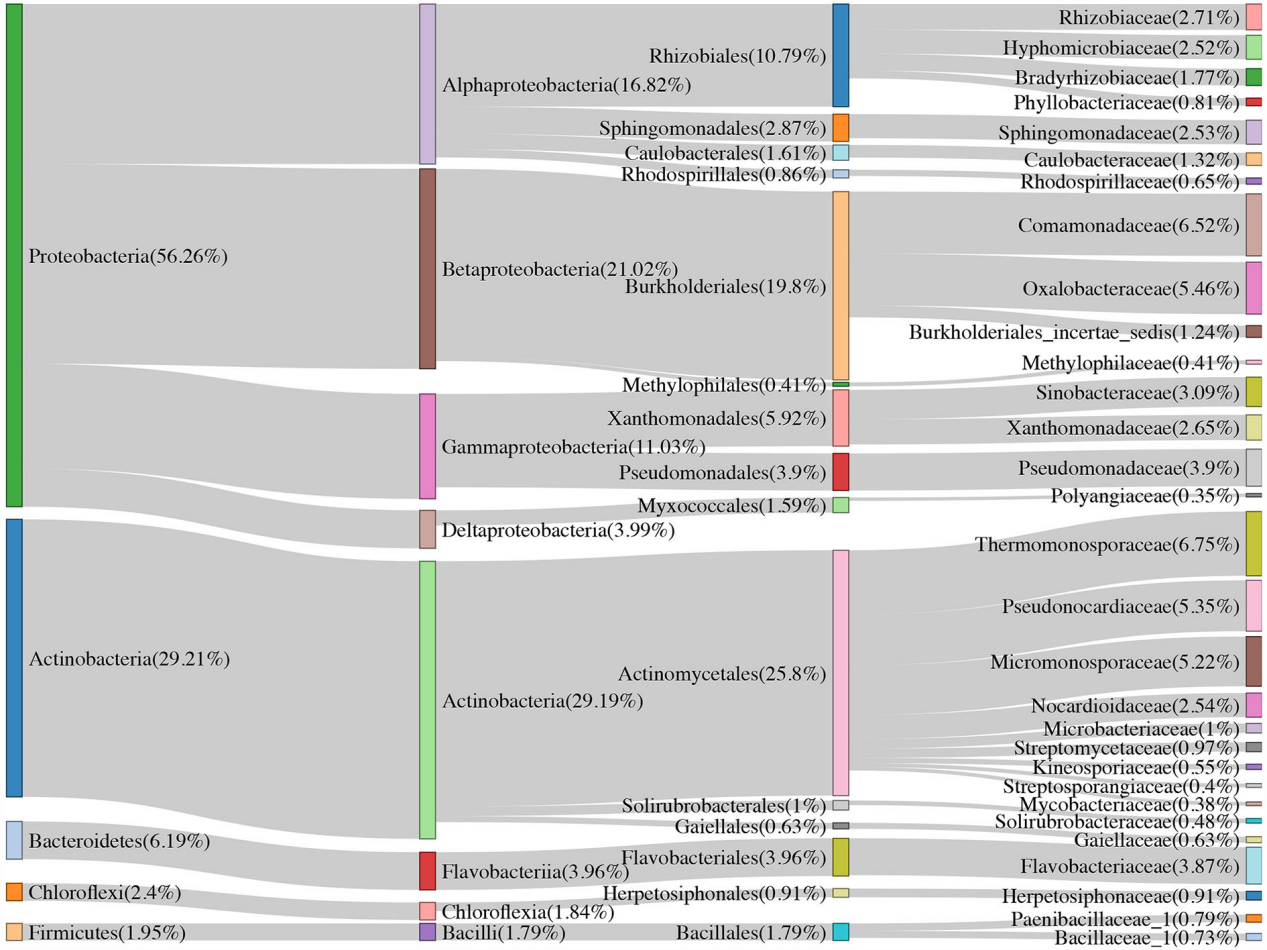
The community composition of Moso bamboo rhizome root microbiota and soil bacteria with their relative abundance list on the right.

Over half of the bacterial sequences were composed of the phyla *Proteobacteria* (56.26%), followed by *Actinobacteria* (29.21%) and *Bacteroidetes* (6.19%) (Figure 2). Soil samples had a higher proportion of *Proteobacteria* (61.55%) than that of *Actinobacteria* (24.23%). However, for the rhizome root samples, the number of sequences from *Actinobacteria* (44.17%) was higher than that from *Proteobacteria* (40.38%). Moreover, combined intensive management reduced the relative abundance of *Proteobacteria, Bacteroidetes, Verrucomicrobia*, and *Nitrospirae* compared to that in the control, whereas the relative abundance of *Actinobacteria* and *Firmicutes* increased.

Approximately 74.51, 52.83, and <50% of the sequences were identified at the family, genus, and species levels, respectively. Therefore, we mainly analyzed the bacterial community at the family and ASV levels. We treated the top 30 families with the highest relative abundance as the core families, which accounted for 93.20% of the total sequences. Apart from unassigned groups, the most abundant family in all 36 samples was *Thermomonosporaceae* (6.75%), followed by *Comamonadaceae* (6.52%). Furthermore, *Thermomonosporaceae* had a contrasting abundance in the soil (0.65%) and root (25.05%) samples. *Flavobacteriaceae* was also abundant in the roots (8.47%), which was not the case in soil samples (2.33%) (Table 2).

**Table 2:** Results of indicator families, core families, and the linear discriminant analysis (LDA) effect size (LEfSe) analysis.

**Core family**	**Soil (%)**	**Root (%)**	**LEfSe family**	**Group**	**LDA value**	***P* value**	**Indicator family**	**Total**	**Indicator value**	***P* value**
**Unassigned** ^b^	30.39 ± 6.38^a^	17.04 ± 10.34	***Bacillaceae***	FM	3.600	0.036*^d^	*Acidimicrobiaceae*	0.15 ± 0.17	0.865	0.013*
***Thermomonosporaceae***	0.65 ± 1.00	25.05 ± 42.00	*Bacteriovoracaceae*	FM	3.596	0.001***	*Alicyclobacillaceae*	0.04 ± 0.04	0.884	0.009**
***Comamonadaceae***	6.64 ± 7.19	6.16 ± 11.09	***Bradyrhizobiaceae***	SM	4.090	0.000***	***Bacillaceae***	0.73 ± 0.54	0.884	0.013*
***Oxalobacteraceae***	6.18 ± 2.49	3.30 ± 2.85	***Burkholderiales***	CK, FM	3.835	0.001***	*Bdellovibrionaceae*	0.28 ± 0.31	0.914	0.001***
*Pseudonocardiaceae*	4.49 ± 4.32	7.94 ± 6.78	***Caulobacteraceae***	CK	4.111	0.000***	***Bradyrhizobiaceae***	1.77 ± 2.61	0.875	0.014*
***Micromonosporaceae***	6.36 ± 5.31	1.81 ± 2.21	*Chitinophagaceae*	CK	3.531	0.000***	***Burkholderiales***	1.24 ± 0.80	0.833	0.001***
*Pseudomonadaceae*	3.86 ± 4.06	4.03 ± 4.17	*Chloroflexaceae*	FM	3.582	0.000***	***Caulobacteraceae***	1.32 ± 1.11	0.888	0.001***
*Flavobacteriaceae*	2.33 ± 2.21	8.47 ± 7.25	***Comamonadaceae***	FM, RR, SM	4.118	0.014*	*Chitinophagaceae*	0.21 ± 0.29	0.869	0.043*
***Sinobacteraceae***	3.49 ± 1.81	1.87 ± 1.26	*Conexibacteraceae*	FM	3.590	0.001***	***Comamonadaceae***	6.52 ± 8.16	0.941	0.001***
***Rhizobiaceae***	2.95 ± 1.51	1.99 ± 1.74	*Erythrobacteraceae*	RR	3.890	0.000***	*Cystobacteraceae*	0.10 ± 0.10	0.922	0.002**
***Xanthomonadaceae***	2.47 ± 1.03	3.19 ± 2.96	***Herpetosiphonaceae***	CK	4.096	0.001***	***Herpetosiphonaceae***	0.91 ± 1.71	0.928	0.015*
***Nocardioidaceae***	2.67 ± 1.41	2.13 ± 1.50	***Hyphomicrobiaceae***	CK, SM	3.916	0.000***	***Hyphomicrobiaceae***	2.52 ± 1.50	0.794	0.004**
***Sphingomonadaceae***	3.27 ± 1.81	0.32 ± 0.37	*Kineosporiaceae*	CK, RR	4.135	0.007**	*Iamiaceae*	0.15 ± 0.22	0.857	0.047*
***Hyphomicrobiaceae***	3.02 ± 1.33	1.01 ± 0.88	*Methylophilaceae*	CK	3.490	0.012*	*Labilitrichaceae*	0.15 ± 0.14	0.907	0.007**
***Bradyrhizobiaceae***	1.99 ± 2.90	1.13 ± 1.36	***Microbacteriaceae***	CK	4.020	0.000***	*Leptospiraceae*	0.20 ± 0.41	0.924	0.027*
***Caulobacteraceae***	1.58 ± 1.09	0.53 ± 0.77	***Micromonosporaceae***	CK, SM	4.251	0.000***	***Microbacteriaceae***	1.00 ± 0.64	0.869	0.001***
***Burkholderiales** ^*c*^*	1.48 ± 0.71	0.54 ± 0.64	*Nocardiaceae*	FM	3.565	0.000***	*Micrococcaceae*	0.23 ± 0.24	0.815	0.031*
***Microbacteriaceae***	1.27 ± 0.47	0.18 ± 0.23	***Nocardioidaceae***	FM	4.051	0.019*	***Micromonosporaceae***	5.22 ± 5.10	0.893	0.001***
***Streptomycetaceae***	1.15 ± 0.59	0.43 ± 0.48	***Oxalobacteraceae***	CK, FM, RR	4.266	0.010**	*Nannocystaceae*	0.10 ± 0.11	0.840	0.037*
***Herpetosiphonaceae***	0.84 ± 0.96	1.11 ± 3.11	*Paenibacillaceae*	FM	3.829	0.009**	*Nitrospiraceae*	0.09 ± 0.16	0.935	0.003**
***Phyllobacteriaceae***	0.89 ± 0.50	0.56 ± 0.55	***Phyllobacteriaceae***	FM	3.578	0.016*	***Nocardioidaceae***	2.54 ± 1.43	0.739	0.032*
*Paenibacillaceae*	0.96 ± 0.85	0.30 ± 0.26	*Pseudomonadaceae*	CK, FM, RR	4.116	0.016*	*Oscillochloridaceae*	0.10 ± 0.11	0.895	0.001***
***Bacillaceae***	0.89 ± 0.51	0.25 ± 0.26	*Pseudonocardiaceae*	FM, RR, SM	4.174	0.008**	***Oxalobacteraceae***	5.46 ± 2.84	0.861	0.001***
*Rhodospirillaceae*	0.46 ± 0.34	1.22 ± 2.01	***Rhizobiaceae***	CK	4.032	0.015*	***Phyllobacteriaceae***	0.81 ± 0.52	0.835	0.002**
*Gaiellaceae*	0.58 ± 0.37	0.78 ± 0.71	*Rhodospirillaceae*	RR	4.192	0.004**	***Rhizobiaceae***	2.71 ± 1.60	0.821	0.001***
*Kineosporiaceae*	0.23 ± 0.23	1.50 ± 1.46	***Sinobacteraceae***	CK, FM	4.237	0.001***	*Rhodocyclaceae*	0.02 ± 0.03	0.898	0.010**
***Solirubrobacteraceae***	0.57 ± 0.31	0.23 ± 0.21	***Solirubrobacteraceae***	SM	3.489	0.000***	*Rubrobacteraceae*	0.04 ± 0.05	0.851	0.014*
*Methylophilaceae*	0.46 ± 0.36	0.26 ± 0.58	***Sphingomonadaceae***	CK, FM	4.183	0.001***	***Sinobacteraceae***	3.09 ± 1.82	0.801	0.003**
*Streptosporangiaceae*	0.27 ± 0.27	0.78 ± 1.68	***Streptomycetaceae***	SM	3.811	0.000***	***Solirubrobacteraceae***	0.48 ± 0.32	0.793	0.003**
*Mycobacteriaceae*	0.41 ± 0.46	0.29 ± 0.22	***Thermomonosporaceae***	CK, RR	4.654	0.020*	***Sphingomonadaceae***	2.53 ± 2.04	0.863	0.004**
			**Unassigned**	CK, FM, SM	3.906	0.006**	***Streptomycetaceae***	0.97 ± 0.64	0.903	0.001***
			***Xanthomonadaceae***	SM	3.584	0.000***	***Thermomonosporaceae***	6.75 ± 22.78	0.990	0.018*
							**Unassigned**	27.05 ± 9.44	0.778	0.001***
							***Xanthomonadaceae***	2.65 ± 1.70	0.806	0.001***

a: Data are presented as mean ± standard deviation (n = 3).

b: Family in bold font indicates the 20 common families shared among indicator species, core microbes, and LEfSe analysis.

c: Burkholderiales is short for Burkholderiales_incertae_sedis.

d: Significance levels: *0.01 < P ≤ 0.05, **0.001 < P ≤ 0.01; ***P ≤ 0.001.

FM: The first treatment was conducted continually with the combined intensive practice for 2 years.

SM: The second treatment which was intensively managed for 3 years, fallowed for the following 3 years, and then highly practiced for another 2 years.

CK: The third treatment was set as a control without any combined intensive management.

RR: Rhizome roots.

**Figure 3:**
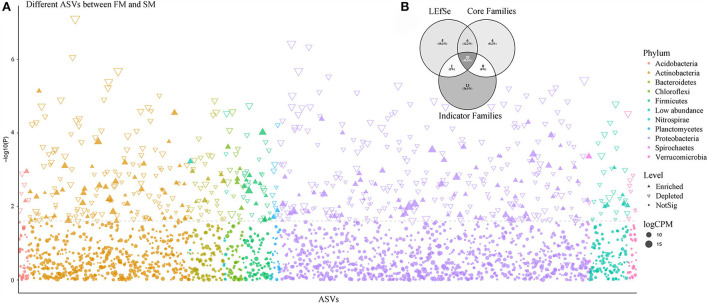
The comparison of core bacterial microbiota predicted by different methods. **(A)** Manhattan plot showing different ASVs enriched in FM and SM. Each dot or triangle represented a single ASV. ASVs enriched in Moso bamboo soil bacteria are represented by filled or empty triangles, respectively (FDR adjusted *P* < 0.05, Wilcoxon rank sum test). ASVs are arranged in taxonomic order and colored according to the phylum. CPM, counts per million. **(B)** The comparison of Moso bamboo core bacteria generated by indicator Families, core Families, and LEfSe *via* Venn diagram.

The LEfSe analysis and Manhattan plot results showed that 160 ASVs belonging to 47 families were enormously enriched in FM, compared to that in SM (Figure 3A). The most abundant ASVs were identified as *Pelomonas puraquae* (ASV2: *Comamonadaceae*), *Bradyrhizobium neotropicale* (ASV15: *Bradyrhizobiaceae*), and *Kibdelosporangium phytohabitans* (ASV69: *Pseudonocardiaceae*), with two unassigned ASVs (ASV36: *Oxalobacteraceae* and ASV17: *Comamonadaceae*). However, the most abundant family was *Xanthomonadaceae*, followed by *Paenibacillaceae* and *Pseudonocardiaceae*. Similarly, 458 ASVs belonging to 69 families were significantly enriched in SM compared to that in FM. The five most abundant ASVs were ASV4, ASV54, ASV52, ASV154, and ASV190. Although these ASVs have not been identified at the species level, they were primarily assigned to *Pseudomonadaceae, Micromonosporaceae, Pseudonocardiaceae*, and *Bradyrhizobiaceae*. The most abundant family was *Micromonosporaceae*, followed by *Nocardioidaceae* and *Sinobacteraceae*. When comparing FM and SM with CK separately, 549 and 453 ASVs were significantly enriched in the first and second intensively managed soils, whereas 601 and 190 ASVs were remarkably depleted, respectively. ASV2 (*P. puraquae*), ASV15 (*B. neotropicale*), and ASV36 (unassigned) were also among the top five most abundant ASVs in FM. The top five most abundant ASVs in SM were ASV9, ASV52, ASV54, ASV130, and ASV154, identified as families *Micromonosporaceae* and *Pseudonocardiaceae*, but none of them were recognized at the species level.

Thirty-four bacterial families and 313 ASVs were identified as indicator taxa with a significance level lower than 0.05. Besides, we examined the common elements among the core family (30), biomarker family (32), and indicator family (34). Twenty families were shared between the three methods (Figure 3B). As previously mentioned, the most abundant family, *Thermomonosporaceae*, was more abundant in roots than in soils. We further found that as the combined intensive management duration increased, the number of bacteria from this family decreased, which was similar to that of *Oxalobacteraceae*, but not *Micromonosporaceae*.

### Relationships between soil properties and soil bacterial abundance in intensively practiced Moso bamboo forests

**Figure 4:**
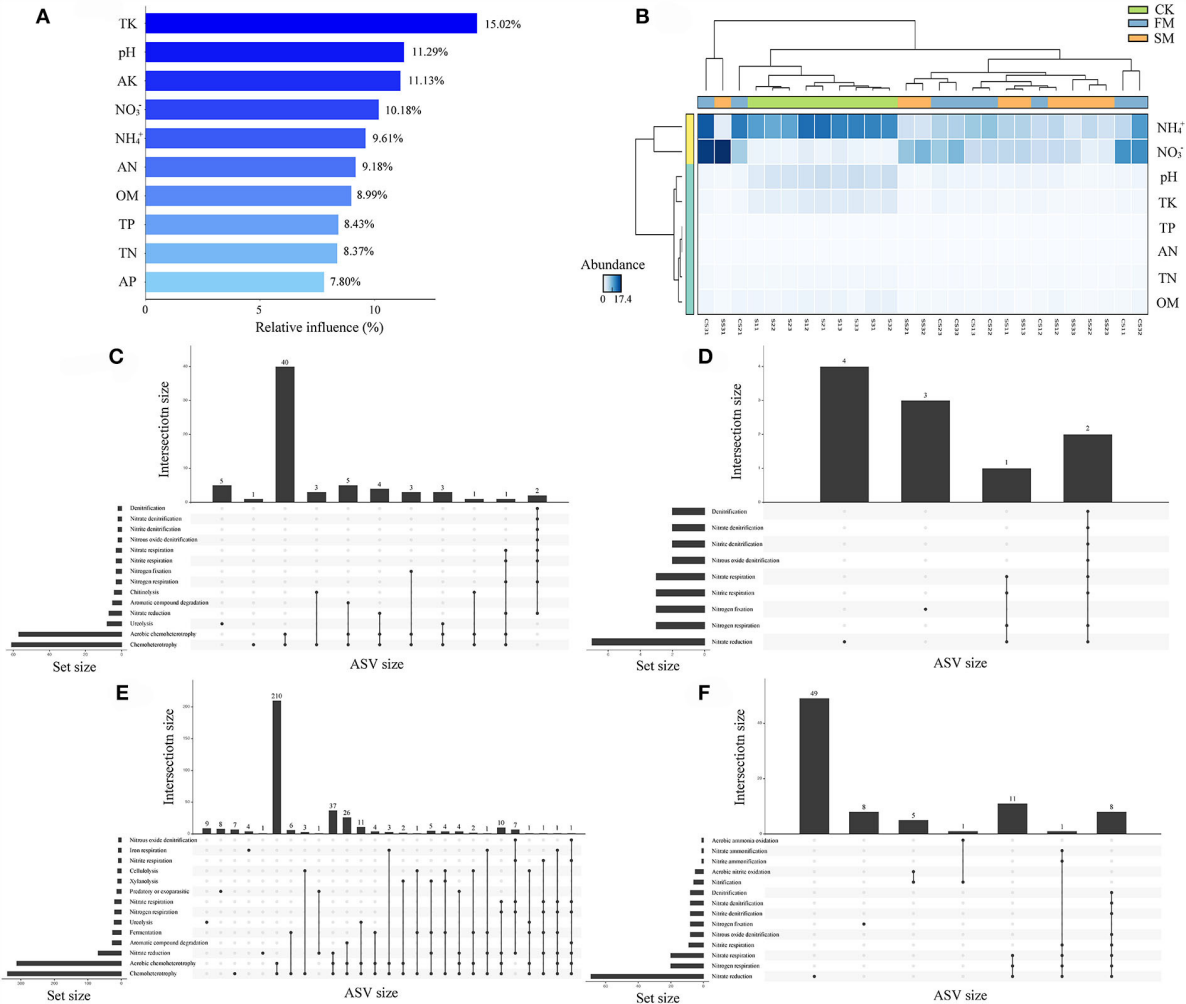
The relationships between soil chemical properties and nitrogen-related functions under combined intensive management. **(A)** The relative influence (%) of predictor variables for Moso bamboo forests was calculated by the aggregated boosted tree model. **(B)** Heatmap revealing the relationship between soil chemical properties and different soil samples. **(C–F)** Upset results show the relationships of nitrogen-related functions on intensively managed soil bacteria predicted by FAPROTAX. The left panel displays the number of ASVs assigned to different functions. Dark circles in the matrix indicate sets of treatments that intersect. **(C)** Abundant ASVs (relative abundance > 0.1%); **(D)** Rare ASVs (relative abundance < 0.01%); **(E)** abundant ASVs related to nitrogen cycles; **(F)** rare ASVs related to nitrogen cycles.

A comprehensive analysis of ABT modeling, Upset, and random forest was performed to dissect the relationships between soil properties and soil bacterial abundance. The ABT modeling uncovered that TK was the primary factor influencing the number of soil bacteria, accounting for approximately 15.02% of the relative influence, followed by pH (11.29%) and AK (11.13%) (Figure 4A). Furthermore, STAMP results indicated that apart from AK and AP, the other eight soil properties significantly affected the soil bacterial community (Kruskal–Wallis H test, *P* < 0.05, Figure 4B). The heatmap also revealed that NH${}_{4}^{+}$ and NO${}_{3}^{-}$ might have different effects on soil bacteria than on other soil properties (Figure 4B). We used FAPROTAX to predict the potential functional capacities of soil bacteria and found that 864 ASVs had ecological functions, of which 164 ASVs were more likely to participate in the N cycling.

**Figure 5:**
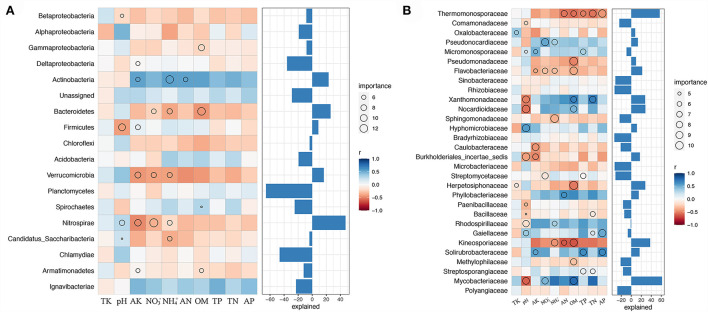
Biological contributions of dominant bacterial phyla **(A)** and families **(B)** for soil nutrient cycling analyzed by random forest.

We examined the biological contributions of dominant bacterial phyla and families to soil properties by random forest (Figure 5). Evidently, not all bacterial phyla contributed equally to various edaphic variables. *Nitrospirae* is an essential variable (explained for nearly 50% of variations) for assessing soil properties, namely, pH, NO${}_{3}^{-}$, NH${}_{4}^{+}$, and AK, indicating its importance in soil nutrient cycling during combined intensive management. *Actinobacteria, Bacteroidetes*, and *Firmicutes* were the most crucial variables for estimating the soil NH${}_{4}^{+}$, OM, and pH, respectively. Furthermore, none of the bacterial communities significantly contributed to AP, TK, TN, and TP (Figure 5A).

Moreover, *Mycobacteriaceae* explained the crucial variation (over 60%) in pH, NO${}_{3}^{-}$, and OM at the family level. *Thermomonosporaceae* and *Kineosporiaceae* were negatively correlated with OM, TP, TN, AP, AN, NH${}_{4}^{+}$, OM, and AN, respectively. Soil TK was mostly explained by the presence of *Oxalobacteraceae* and *Herpetosiphonaceae*, NO${}_{3}^{-}$ by *Pseudococardiaceae, Flavobacteriaceae, Streptomycetaceae*, and *Mycobacteriaceae*, and NH${}_{4}^{+}$ by *Pseudococardiaceae, Flavobacteriaceae, Sphingomonadaceae, Rhodospirillaceae*, and *Kineosporiaceae* (Figure 5B).

To learn more about the effect of N-related functions on intensively managed soil bacteria, abundant (relative abundance > 0.1%), rare (relative abundance < 0.01%), and N-related ASVs from FAPROTAX results were compared and analyzed using Upset plots (Figures 4C–F). For abundant ASVs, the most dominant function was “Chemoheterotrophy.” Further, N cycling-related pathways such as “Nitrate reduction,” “Nitrogen respiration,” and “Nitrogen fixation” were among the first 14 pathways (Figure 4C). A similar result was observed for rare ASVs (Figure 4D). Next, we compared abundant and rare ASVs primarily related to N cycles. The results showed that the N cycling-related abundant ASVs were mainly involved in “Nitrate reduction,” “Nitrogen respiration,” “Nitrogen fixation,” “Nitrite respiration,” “Nitrate respiration,” “Nitrous oxide denitrification,” “Nitrite denitrification,” “Nitrate denitrification,” and “Denitrification” (Figure 4E). Furthermore, for N cycling-related rare ASVs, “Nitrification,” “Aerobic nitrite oxidation,” “Nitrite ammonification,” “Nitrate ammonification,” and “Aerobic ammonia oxidation” were uniquely identified, apart from the nine pathways found in N cycling-related abundant ASVs (Figure 4F).

### Potential function variations of soil bacteria after combined intensive management

**Figure 6:**
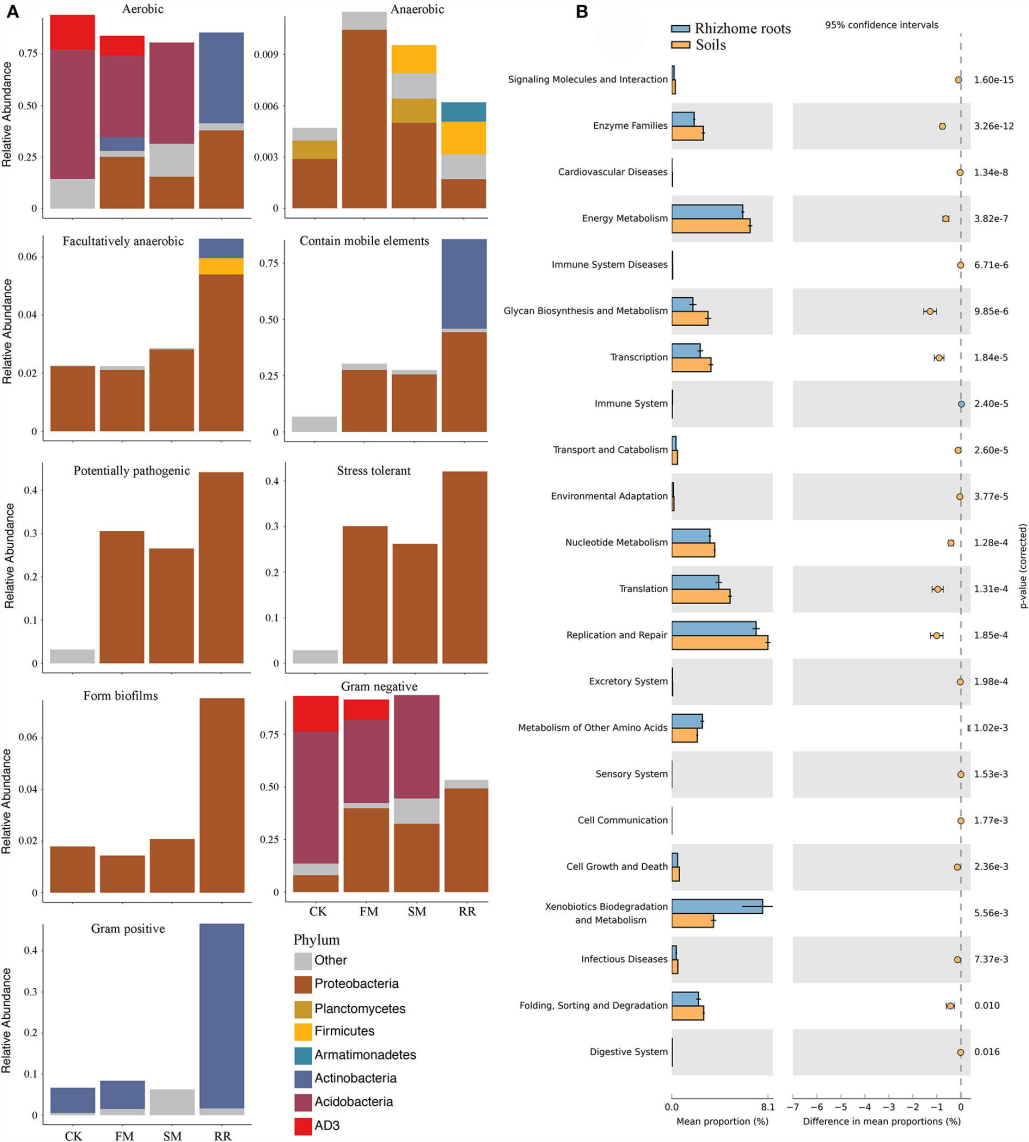
Biologically interpretable phenotypes and functional analysis of Moso bamboo-associated bacteria after combined intensive management. **(A)** Biological phenotypes predicted by BugBase; **(B)** Significantly different pathways between rhizome roots and soil bacterial microbiota at KEGG functional category 2 with their statistical significance estimated in STAMP.

While practicing combined intensive management, the relative abundance of aerobic bacteria decreased, anaerobic bacteria increased, and facultative bacteria remained relatively stable. Furthermore, the relative abundance of bacteria containing mobile elements was higher in FM and SM than that in CK. Notably, after combined intensive management, soil bacterial function in pathogenic and stress tolerance increased significantly. Comparing functions in different samples suggested that bacteria containing mobile elements, forming biofilms, potentially pathogenic, and stress tolerance were more abundant in root samples than in soil samples. In contrast to soil samples, an increase in Gram-positive bacteria was observed in root samples compared to that of Gram-negative bacteria (Figure 6A).

PICRUSt revealed that 119 of the 301 annotated pathways significantly differed between CK, FM, SM, and RR (corrected *P* < 0.05). Most of the annotated ASVs were related to “Metabolism” in the level 1 category, followed by “Genetic information processing.” In the level 2 category, 29 of the 37 predicted metabolic pathways differed significantly among different sample types (Welch's *t-*test corrected by Bonferroni, *P* < 0.05). Most of them were related to “Amino acid metabolism,” such as “Amino acid related enzymes,” “Alanine, aspartate and glutamate metabolism,” and “Glycine, serine and threonine metabolism.” Some were related to “Carbohydrate metabolism,” namely, “Amino sugar and nucleotide sugar metabolism,” “Starch and sucrose metabolism,” and “Pyruvate metabolism.” When considering the soil and root bacteria, 22 of the 35 predicted metabolic pathways in the level 2 category differed significantly between rhizome roots and soil microbiota (Welch's *t*-test corrected by Bonferroni, *P* < 0.05). Among the significantly different metabolic pathways, “Replication and repair,” “Xenobiotics biodegradation and metabolism,” “Translation,” “Energy metabolism,” and “Nucleotide metabolism” were relatively more abundant than the remaining categories (Figure 6B).

## Discussion

Moso bamboo forests, possessing unique biological characteristics such as perennial, active clonal reproduction, and a highly complicated below-ground rhizome system, differ significantly from agriculture and common forestry ecosystems. The results of the combined intensive management of soil properties and bacterial microbiota of Moso bamboo forests will help fill gaps in forestry ecosystem research and provide guidelines for forestry management and sustainable development.

### Effect of combined intensive management on the diversity of bacterial communities

The decreased diversity and richness of soil bacteria following combined intensive management in the present study indicates that these management practices adversely affected Moso bamboo soil bacterial communities, which is consistent with the previous findings of Chen et al. (2019), who also found a reduction in bacterial diversity and community composition after long-term intensive management in a bamboo forest; the reduction was partially attributed to the altered soil pH and NO${}_{3}^{-}$. The fertilization-induced decrease in soil pH and accumulation in NO${}_{3}^{-}$ resulted in soil acidification in bamboo forests, providing unsuitable habitats for most soil bacteria.

In this study, TK was identified as the most critical driver of the soil bacterial microbiota in the Moso bamboo forest, followed by pH and AK. The TK content increased in response to FM. After SM, the TK content decreased and was even lower than that in the un-mulched soils, revealing that soil bacteria may have a high demand for TK. The decrease in the soil pH was higher with FM than that with CK. After fallowing for 3 years with SM, the soil pH increased and was comparable to that of CK, implying that moderate fallow contributed to the restoration of soil pH. The contents of the remaining eight factors (AK, AN, AP, NH${}_{4}^{+}$, NO${}_{3}^{-}$, OM, TN, and TP) continuously increased with the combined intensive management, revealing nutrient accumulation due to fertilization during forest management.

### Excessive accumulation of nutrient resources in intensively managed soils has redesigned bacterial communities of Moso bamboo forests

Combined intensive management also potently affected the bacterial communities in Moso bamboo soil (Figure 1F). The relative abundance of the phyla *Actinobacteria* and *Firmicutes* increased, while *Proteobacteria, Bacteroidetes, Verrucomicrobia*, and *Nitrospirae* decreased following combined intensive management. The relative abundance of *Chloroflexi* decreased in FM but was restored to the CK level after lying fallow for 3 years. Studies conducted in agricultural fields have frequently reported that intensive management reduces bacterial abundance and diversity, as well as leads to significant changes in the community structure (Ai et al., 2018; Chen et al., 2019).

Changes in bacterial community composition after intensive management can be explained by the copiotrophic hypothesis (Fierer et al., 2012; Ramirez et al., 2012). The copiotrophic groups thrive under nutrient-rich conditions, while oligotrophic groups survive best in low-carbon environments (Yu et al., 2019). *Actinobacteria* are fast-growing copiotrophic microorganisms that rely on labile carbon sources and high nitrogen amendments (Wang et al., 2020b). A large amount of nutrients in the soil obtained from fertilization during combined intensive management provided metabolic substrates for the activity of copiotrophic microbes. Tillage improves soil aeration and microbial access to organic matter (Chen et al., 2019). These practices led to the enrichment of copiotrophic *Actinobacteria via* increased oxygen levels and pressures from other heterotrophs. Zheng et al. (2018a) reported that mulching increased the relative abundance of *Actinobacteria* and *Firmicutes* in a semiarid orchard system. Furthermore, straw incorporation induced copiotrophic bacterial growth in a rice-wheat cropping system (Zhao et al., 2017). Alternatively, *Bacteroidetes* and *Nitrospirae* are often considered oligotrophic (Davis et al., 2011; Fierer et al., 2012), especially the comammox *Nitrospira*, which prefers acidic soils and nitrogen-depleted environments (Li et al., 2019). *Chloroflexi* may also be copiotrophic because they decompose major organic matter. High carbon levels induced by organic matter decomposition result in colonization (Freeman et al., 2009; Eisenlord and Zak, 2010). Although *Proteobacteria* are considered copiotrophic, their relative abundance did not increase but decreased after combined intensive management, which is consistent with previous findings in a subtropical bamboo forest (Chen et al., 2019). Because *Proteobacteria* are taxonomically and functionally diverse, it is possible that their resistance to soil disturbance and adaptation to altered edaphic chemical factors consistently contributed to the abundance of *Proteobacteria*.

### Functional capability stability of microbiota in response to combined intensive management

Currently, significant differences in the functional capabilities of rhizome roots and soil bacteria in response to combined intensive management have been observed (Figure 6B). Functional capabilities involved in metabolic pathways, such as “Energy metabolism,” “Nucleotide metabolism,” “Glycan biosynthesis and metabolism,” and “Replication and repair” were specifically enriched in the soil bacterial microbiota. In contrast, root bacteria are exclusively abundant in “Xenobiotics biodegradation and metabolism.” The soil bacteria-abundant function, the so-called broad functioning (e.g., “Energy metabolism,” “Nucleotide metabolism,” “Glycan biosynthesis and metabolism”), was widely distributed, whereas, root bacteria-enriched function, “Xenobiotics biodegradation and metabolism,” was considered as specialized function and usually conducted by particular groups of organisms or tissues (Delgado-Baquerizo et al., 2016). The enrichment of “Xenobiotics biodegradation and metabolism” in root samples partly reflects the metabolic resistance of the host to pesticides. As soils and roots serve as different niches, they selected functionally different taxa, rather than taxonomic attributes, as their bacterial partners lead to the differentiation of functional capabilities (Chen et al., 2020).

When considering the effects of combined intensive management and fallow on soil metabolic functions, we found that functions related to “Amino acid metabolism,” “Glycan biosynthesis and metabolism,” “Biosynthesis of other secondary metabolites,” “Cell growth and death,” etc. decreased in response to combined intensive management. In contrast, “Xenobiotics biodegradation and metabolism” increased, but these changes were unremarkable. Similar results have been reported in long-term fertilized Moso bamboo forests, where the metabolic functions of soil bacterial communities did not change significantly in response to intensive management practices, namely, understory removal, soil tillage, and annual fertilization (Chen et al., 2019). Broad functioning is reportedly insensitive to changes in bacterial diversity and community composition, although this is primarily driven by nutrient availability (Delgado-Baquerizo et al., 2016). The fertilization applied in this study provided abundant nutrients for soil bacteria, partly explaining the relative stability of the functional capabilities of soil bacteria, regardless of practice duration. Furthermore, fallow did not influence the bacterial function capability.

### Active denitrification despite the detection of comammox *Nitrospirae*

Nitrogen metabolism is a specialized function in soil environments that contributes significantly to global nitrogen cycling. Bacteria capable of nitrogen metabolism are often found in rice and other crop systems and provide nitrogen for plants. The loss of soil bacterial diversity significantly reduced the potential denitrification activity (Philippot et al., 2013) and pesticide mineralization capacity (Singh et al., 2014). Accordingly, the reduced bacterial diversity found in this study could influence the soil-specialized functions. Concordantly, nitrogen metabolism-related functions, such as nitrate reduction, nitrogen respiration, nitrogen fixation, and nitrite respiration, were enriched in Moso bamboo forest soils. Dissimilatory nitrate reduction to ammonium (DNRA), denitrification, anaerobic ammonium oxidation (anammox), and biological nitrogen fixation (BNF) influence nitrogen use efficiency in rice production (Pandey et al., 2021). Notably, DNRA and BNF contribute to the nitrogen supply, whereas denitrification results in nitrogen loss. Continuous nitrogen fertilization in rice paddies increases denitrification and reduces N_2_ fixation (Pandey et al., 2019). Dissimilatory nitrate reduction to ammonium rather than denitrification dominates nitrate reduction in subtropical pasture soils upon rewetting.

Previous research has revealed that the intensive management of Moso bamboo forests altered soil nitrifying and denitrifying microorganism communities controlled by numerous factors. The main factor influencing the diversity and composition of nitrifiers was soil nitrogen, whereas denitrifiers were predominantly affected by soil organic carbon (SOC) and AK. Currently, comammox *Nitrospirae* has been detected in Moso bamboo forests. The phylum *Nitrospirae*, a complete ammonia oxidizer that can oxidize NH${}_{4}^{+}$ to NO${}_{3}^{-}$ in a single organism, is critical for nitrogen cycling, especially in the nitrification process (Li et al., 2019). A single *Nitrospira* species expresses ammonia monooxygenase and hydroxylamine dehydrogenase, which largely contribute to complete nitrification (Daims et al., 2015; van Kessel et al., 2015). Due to its complete ammonia oxidizer properties, *Nitrospirae* prefers to use ammonia as the substrate for growth in soils and are especially sensitive to nitrite concentrations and soil acidity (Han et al., 2017). With the implementation of combined intensive management, the relative abundance of *Nitrospirae* decreased in the current study. The increase in the accumulation of NO${}_{3}^{-}$ and the decrease in soil pH resulting from fertilization may be unfavorable for *Nitrospirae*. Nitrification is aerobic; however, mulching-induced soil hypoxia creates an adverse environment for these soil bacteria. All these factors led to a gradual decline in *Nitrospirae* populations.

Alternatively, an excessive amount of NO${}_{3}^{-}$ leads to denitrification, such as nitrate reduction. The activity of denitrifying bacteria in long-term intensively managed Moso bamboo forests has been uncovered, with the abundance of denitrifying genes (*nir*K, *nir*S, *nos*Z) increasing or remaining stable after management (Cao et al., 2020). It has been suggested that management-induced increases in SOC, TN, and AN were more strongly correlated with functional gene abundance than decreases in soil pH. Simek et al. (2000) also reported that AK had a highly significant positive correlation with denitrification potential. Although we did not check the SOC content, the accumulation of AN, TN, and AK, and especially the NO${}_{3}^{-}$ provided nutrients for denitrifying bacteria, ensuring their functioning in denitrification. The ABT model also supported that AK, NO${}_{3}^{-}$, and AN were responsible for 30.49% of the relative influence of ASV abundance, which contributes to nitrogen metabolism. From a taxonomic perspective, most denitrifying bacteria belonging to *Proteobacteria* and *Proteobacteria* were remarkably enriched in soils compared to that in roots in the current study. Different bacteria respond differently to combined intensive management; therefore, further research is required to determine the exact mechanisms underlying nitrogen metabolism.

## Conclusion

This study demonstrated that combined intensive management applied in Moso bamboo forests lowered the bacterial diversity of soils and rhizome roots but had a slight impact on community composition. The decrease in bacterial diversity and abundance can be explained by intensive management-induced changes in soil properties. Deep tillage, fertilization, and organic material covering significantly increased the AK, AN, AP, NH${}_{4}^{+}$, NO${}_{3}^{-}$, OM, TN, and TP contents in soils but markedly decreased the pH value. The relative stability of community composition and functional capacity could result from species–species interactions in soil bacterial communities for the equilibrium of the soil ecosystem, which needs further investigation. The excessive accumulation of soil nutrients redesigned the bacterial community composition, potentially influencing microbial functions involved in soil N cycling. We concluded that intensive management-induced changes in soil properties reshape the bacterial community structure, leading to the differentiation of functional capacities. This study sheds light on the bacterial effects of intensively managed Moso bamboo forests, as well as provides theoretical support for the rational application of combined intensive practices in forestry ecosystems. These results hold paramount significance for improving soil quality and fertilizer efficiency and further promoting local economic development.

## Data availability statement

The datasets presented in this study can be found in online repositories. The names of the repository/repositories and accession number(s) can be found below: https://www.ncbi.nlm.nih.gov/, PRJNA692804.

## Author contributions

YZ conducted the experiments, analyzed the data, and wrote the manuscript. XinzL and WZ conducted the experiments. QS and XincL conceived the experiments. YC revised the manuscript. All authors read and approved the final manuscript.

## Funding

This work was supported by the National Natural Science Foundation of China [31901368 to YZ].

## Conflict of interest

The authors declare that the research was conducted in the absence of any commercial or financial relationships that could be construed as a potential conflict of interest.

## Publisher's note

All claims expressed in this article are solely those of the authors and do not necessarily represent those of their affiliated organizations, or those of the publisher, the editors and the reviewers. Any product that may be evaluated in this article, or claim that may be made by its manufacturer, is not guaranteed or endorsed by the publisher.
